# Factors Affecting Direct and Transfer Entrants’ Active Coping and Satisfaction with the University

**DOI:** 10.3390/ijerph17082803

**Published:** 2020-04-18

**Authors:** Kin Cheung, Jeremy Ng, Hilda Tsang, Kelvin K. L. Pang, C. L. Johnny Wan, Kristin Moser

**Affiliations:** 1 School of Nursing, The Hong Kong Polytechnic University, Yuk Choi Road, Hung Hom, Kowloon, Hong Kong, China; jeremy.ng@polyu.edu.hk (J.N.); hilda.ht.tsang@polyu.edu.hk (H.T.); 2 Department of Logistics and Maritime Studies, The Hong Kong Polytechnic University, Yuk Choi Road, Hung Hom, Kowloon, Hong Kong, Chinajohnny.wan@polyu.edu.hk (C.L.J.W.); 3 Office of Institutional Research & Effectiveness, The University of Northern Iowa, 104 Seeley Hall, Cedar Falls, IA 50614, USA; kristin.moser@uni.edu

**Keywords:** psychological wellbeing, active coping, adjustment, transition, university students, community college transfer students

## Abstract

Psychological wellbeing is vital to public health. University students are the future backbone of the society. Direct and transfer entrants might encounter different adjustment issues in their transition from secondary school or community college to university studies. However, worldwide, the factors affecting their active coping and satisfaction with the university are currently unknown. The purpose of this study was to address this gap. Nine-hundred-and-seventy-eight direct entrants and 841 transfer entrants, recruited by convenience sampling, completed a cross-sectional survey study in 2018. A valid and reliable Hong Kong modified Laanan-Transfer Student Questionnaire (HKML-TSQ) was used to collect data. Multiple methods of quantitative data analysis were employed, including factor analyses, test of model fit, t-tests, correlations, and linear regression. The results showed that the transfer entrants had relatively less desirable experiences in their adjusting processes than did the direct entrants. There was evidence of both common and different factors affecting the two groups’ active coping and satisfaction with the university. Different stakeholders from community colleges, universities, and student bodies should work collaboratively to improve students’ transitional experiences before, during and after admission to the university.

## 1. Introduction

The common pathways to university studies are through post-secondary admission [known as direct entrance] and community college transfer [known as vertical transfer entrance] [1,2]. Students from different pathways to university studies might experience different adjustment issues, which might affect their psychological wellbeing. However, most studies have investigated students’ university experiences solely from the perspectives of individual groups (i.e., direct entrants (DEs) [3] or transfer entrants (TEs) [4,5]), but not many have compared the two. Studies involving both groups of students have focused mostly on their academic performances [4,6,7,8,9,10,11,12]. Although the findings have been inconsistent, TEs have been shown to have similar, or even higher, grade point averages (GPAs) than DEs at graduation [4,13]. This could, however, be a result of the students’ GPAs not accounting for the grades of subjects of which the credits have been transferred [4]. Nonetheless, adjustment to the new environment (i.e., university) can affect students’ academic and social involvement differently. For instance, some studies have found the “transfer shock” phenomenon [4,14] in TEs. Besides, the term “campus culture shock” has been used to describe TEs’ struggles with the new and unfamiliar university campus culture [13,15]. Compared with DEs, TEs have been found to have higher study loads [4], more mental health problems [13], and higher attrition rates [7]. Another problem that has been identified in TEs is related to their integration, engagement and adaptation in university, which are thought to be tied strongly to academic success [10]. While better adjustment to university life can be mediated by more use of active coping, less use of avoidance coping, and active seeking of social support [16], it remains unclear whether the factors affecting these coping strategies differ between students with different routes of entry. In a study using the National Survey of Student Engagement, TEs were found to engage less than direct entrants [1]. In our ongoing literature review of 29 studies of both groups of entrants, there were only two that examined the social adjustment of TEs, but neither considered this in relation to academic adjustment. One of these [10] did not yield generalizable findings since only final year engineering and computer science students were investigated, and their social activities were limited to sororities and fraternities, community service groups, spiritual groups, sports and clubs. The other study, conducted by Wang and Wharton [12], found that TEs participated less in social activities such as campus or student organizations, and made less use of student support services. To fill the research gap and contribute to the literature, this study explored and compared the two groups’ experiences of academic and social adjustment in their university studies, with the goal of identifying the factors affecting their active coping and satisfaction with the university [13,17].

### 1.1. Theoretical Framework

**Figure 1:**
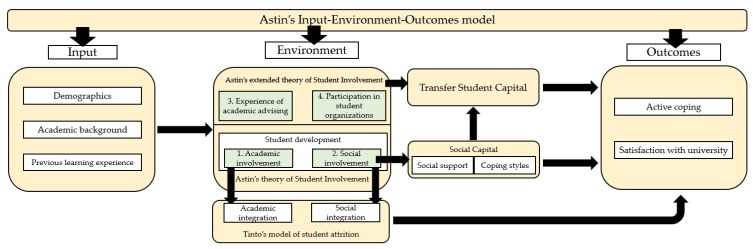
Theoretical framework for this study.

This study was guided by a synthesis of various notable theories for conceptualizing the factors that might affect the direct and TEs’ active coping and satisfaction with university. Figure 1 presents the theoretical framework. Astin’s Input–Environment–Outcomes (IEO) model [18] is adopted frequently to explore the impacts of university study on students. Input is defined as students’ characteristics at the time of entry to university, that can be operationalized as demographics, academic backgrounds, and previous learning experiences [19].

The Theory of Student Involvement [18] has been adopted to explain how the environment, as defined in Astin’s IEO model, can influence student development. Others have found this theoretical perspective useful for studying students’ academic and social adjustment processes [20]. Both the original [18] and updated [12] versions of the theory entail students’ academic and social involvement. According to Tinto’s model of student attrition [21], which has been applied to studies of transfer students (e.g., Getzlaf et al. [22]), integration into the academic and social systems of university leads to an increased level of commitment to university study [23] and enhanced quality of student persistence in learning [24]. Students’ social involvement can also contribute to their social capital.

The notion of social capital was first proposed by Bourdieu [25] and is considered to be “one of the most influential concepts in sociology” (p. 279) [26]. It has been deployed in a diversity of contexts including higher education [27]. The concept of social capital refers to the presence of one’s “institutional relationships of mutual acquaintance and recognition”, or one’s membership of a group (p. 286) [25]. Social capital can be in the form of social networks from which individuals can draw upon social support [28] which, along with coping styles, also appear as newly added constructs in the updated version of the transfer student capital model [29,30]. Social capital not only offers educational benefits, but also facilitates the pursuit of social outcomes during the process of attaining a certain status [31], for example, social adjustment to university study.

The transfer student capital model, applying to both DEs and TEs, involves a host of factors in bringing about successful transition to university [30]. The model refers to the process by which students acquire the knowledge, skills and experience needed to achieve success at the university [20]. Added to this model are the four dimensions identified in an extended version of Astin’s theory of student involvement: experience of academic advising (conceptualized as their use of university support services), academic involvement, social involvement, and participation in student organizations [12]. These four dimensions of students’ undergraduate experiences can be considered environmental variables according to Astin’s IEO model [18]. These concepts and theories serve as the theoretical foundation of various constructs in the modified Laanan-Transfer Students’ Questionnaire (L-TSQ and ML-TSQ) to measure the factors affecting transfer students’ active coping and satisfaction with the university [29,30], and can be considered as outcome variables under Astin’s IEO model [18].

### 1.2. Differences in Adjustment Experiences between DEs and TEs

Both DEs and TEs encounter challenges in their academic and social integration into university [10], such as large class sizes (DEs [32]; TEs [4]); impersonal organizational structures (DEs [33]; TEs [4]); the need to learn to exercise more self-discipline than in secondary school, adjustment to new learning styles [10]; and mental health issues (DEs [34]; TEs [13]). However, the adjustment experiences of the two groups of students can differ due to their different routes of entry. DEs might be less mature at the time of admission but they have 4 years to acclimatize to the university culture. On the other hand, even though TEs arrive with some post-secondary education experience from their community colleges, they have shorter study periods (i.e., most with two years) at university [5]. In recognition of their prior learning, TEs can often be given credit transfer for some subjects at the junior level, meaning that they can start their university studies by enrolling straight in senior subjects. In these classes, they are newcomers to the cohort of DEs who have already been acquainted for 2 years [13]. In terms of social integration, there might be insufficient interactions between the two groups of students [4]. As a consequence, TEs might find it difficult to make new friends [13]. If they are unable to join communities such as study groups, their academic outcomes might be hampered [35]. Furthermore, DEs are more likely to have known the teachers and to have adapted to the learning styles [36]. A further major difference between the two groups is that DEs have been found to receive more attention in various aspects such as orientation and counselling [4,17]. These differences in adjustment experiences, serving as part of the holistic university experience, could give rise to different coping styles or strategies [36] and levels of satisfaction with the university [37]. 

In summary, the majority of prior studies comparing DEs and TEs have focused on their academic performances, with less attention given to their psychosocial adjustment experiences. Moreover, research on TEs has been conducted mostly in western contexts, where student populations are demographically more diverse [30]. There is a scarcity of studies investigating TEs in Asian contexts. Therefore, the objectives of this study were twofold: (1) to explore the similarities and differences between TEs and DEs in their perceptions of university experiences, particularly in terms of their academic and social adjustments, and (2) to identify the factors affecting their active coping and satisfaction with the university. These questions are important because universities have a responsibility to provide socially supportive environments to all students, regardless of their entry routes.

## 2. Methods

### 2.1. Research Design and Context

This was a cross-sectional survey study using a mix of convenience and snowball sampling. Ethical approval for conducting the institution-wide survey (HSEARS20180104005-01) was obtained from the Institutional Review Board. All full-time undergraduate students from one local university in Hong Kong were invited via email, posters and in-class promotion to fill in an online questionnaire between April and November 2018. Local students who had been admitted to university from both secondary schools and community colleges were included.

In this study, the DEs were those admitted from secondary schools and completing their undergraduate study in the normal duration of 4 years, while TEs were those admitted from local community colleges. However, some DEs had finished either 1-year or 2-year community college or university study—their study durations and the resources they received were the same as for students admitted from secondary school. In Hong Kong, a quota (i.e., a certain number of places) is assigned to the government-funded universities to accommodate TEs to complete their undergraduate studies within 2 years (hereafter referred to as 2yTEs). It is a noteworthy common practice in Hong Kong that these 2yTEs are largely fresh graduates of local community colleges.

### 2.2. Instrument: The HKML-TSQ Questionnaire

The modified Laanan-Transfer Student Questionnaire (ML-TSQ) [29] was adapted and employed in this study with the permission of its author. A range of tests was performed on the original ML-TSQ to establish its content validity, construct validity and reliability, and to examine the relationships between the independent and the dependent variables [29]. The internal consistencies of the constructs ranged from 0.74 to 0.94 [30]. For this study, the adapted version of the ML-TSQ (hereafter HKML-TSQ) was reviewed and refined, first by a panel of eight local educational research experts, and then a panel of nine local and overseas experts. In this version, some items were modified to fit the local context. A content validity index (CVI) of 0.99 was found, which was higher than the standard acceptable level of 0.75 [38]. For the appropriateness and readability, 11 local undergraduate students were invited to fill in the questionnaire. Minor revisions were made to some wording.

The HKML-TSQ consisted of: (a) items eliciting students’ socio-demographic information (e.g., year of birth, gender, year of intake); (b) 8 items on their perceptions of the university (renamed to perceived disparity: transfer vs non-transfer students); (b) 10 items on processes of adjusting to university life; (c) 22 items on satisfaction with the university (renamed to university support), and one item on overall university experience; (d) 15 items on coping style at the university, and (e) 10 items on social support at the university. A 5-point Likert scale (1 = completely disagree and 5 = completely agree) was used to assess the students’ levels of agreement with each item, except for the items on university support, which were rated on a 4-point Likert scale (1 = very dissatisfied, 4 = very satisfied) to avoid any central tendency.

### 2.3. Data Analysis

Exploratory factor analyses (EFA) were conducted for each construct by using the general rule of an eigenvalue > 1 [39]. The maximum likelihood extraction method and oblimin rotation were used. Kaiser-Meyer-Olkin (KMO) tests were conducted to measure the sampling adequacy. Cronbach’s alpha reliability statistics were used to test for the scales’ internal consistency.

The tolerance values and the variance inflation factor (VIF) were computed to examine the multicollinearity among the independent variables included in the analysis. Confirmatory factor analyses (CFA) were conducted on the original study factors and the new factors emerged from EFA. The chi-square test of model fit, the goodness-of-fit index (GFI), the comparative fit index (CFI), the Tucker-Lewis index (TLI), and the root mean square error of approximation (RMSEA) were applied to assess the fit of the model.

Independent samples T-tests were used to investigate the differences between the scores of the DEs and 2yTEs on the scales and on the individual items measuring perceived disparity, process of adjusting to university, university support, coping style at university, and social support received. 

Pearson’s correlation test was used to test the correlations between the scales. Variables with statistically significant correlations with student coping and student satisfaction were selected for linear regression analysis (forward) to explore the strongest predictor of the two factors. SPSS analytical software version 25 was used for the data analysis. CFAs were performed with SPSS AMOS 25 (IBM Corp., Armonk, NY, USA).

### 2.4. Ethical Approval 

Ethical approval was obtained from the Human Subjects Ethics Sub-Committee of the Hong Kong Polytechnic University (HSEARS20180104005-01). 

## 3. Results

### 3.1. Student Demographics

There were 1819 respondents, comprising 841 (46.2%) 2yTEs and 978 (53.8%) DEs. The students represented all 28 academic departments of the university. The sample consisted of 34% male and 66% female, aged between 19 and 52 years (mean = 21.6, SD = 1.92). Most of the participants were in the third (35.3%) or fourth (28.2%) year of study, with the others distributed across first (17.6%), second (12.5%), and fifth years (6.3%). It is noteworthy that all 2yTEs were admitted to the university as junior-year students, which is comparable to DEs in their third year of study. As shown in Table 1, these data were consistent and comparable with the university-wide data.

### 3.2. Factor Analysis

For perceived disparity: transfer vs non-transfer students, two factors were loaded and accounted for more than 42.00% of the total variance, among 2yTEs and DEs (Table 2), which were different from the original study [29] with one factor.

Two factors were loaded and accounted for the process of adjusting to university (Table 3), and accounted for more than 31.00% of the total variance. This was similar to the original study with two factors.

For university support, three factors were loaded and accounted for more than 47% of the total variance, among 2yTEs and DEs (Table 4). These were different from the original study with two factors. 

Four factors were loaded for coping style and accounted for more than 53% of the total variance (Table 5), similar to the original study with four factors. Similarly, two identical factors were loaded and accounted for more than 47% of the total variance for social support at the university, among 2yTEs and DEs (Table 6), which were similar to the original study with one factor.

The internal consistencies of altogether 13 factors were calculated using Cronbach’s alpha and were found to be acceptable (Table 7).

### 3.3. Differences between Two Groups of Students on Their Experience and Perceptions

As the results of factor analyses between the two groups of students were similar, the factor structure that emerged from the whole data was used. Table 8 shows the comparison of experience and perceptions between the two groups of students. Results of t-tests yield that the perceptions of DEs scored statistically significantly higher for social adjustment; all the university support factors (i.e., general support and advising, academic experience and advising, institutional attributes, and overall satisfaction on university experience); coping style: active and social; and social connections.

The perceptions of 2yTEs scored statistically significant higher (*p* < 0.0001) than the DEs in transition adjustment (with items “drop in GPA” and “increase in stress”) and perceived greater disparity in university resources (higher poor perceptions). 

Comparisons at the item level revealed that the DEs had better adjustments to the academic standards (*p* < 0.05) and social environment (*p* < 0.01), received more university support (*p* < 0.05) at the university, while the 2yTEs experienced a heavier study load (*p* < 0.001), a drop in their academic performance (*p* < 0.001), felt stigmatized (*p* < 0.001), received insufficient resources and support (*p* < 0.001), and had less opportunities for overseas exchanges (*p* < 0.001) (results not shown).

### 3.4. Correlations among Independent and Dependent Variables

In both groups, student coping and student satisfaction were positively and significantly correlated with most of the variables, including social adjustment; general support and advising, academic experience and advising; institutional attributes; emotional (coping style); social connections; and sense of belonging, with correlation coefficients R ranging from 0.266 to 0.586 (*p* < 0.01) for 2yTEs and 0.227 to 0.531 (*p* < 0.01) for DEs.

However, 2yTEs’ overall university satisfaction correlated mildly negatively and significantly with academic study (R = −0.175 at *p* < 0.01) and transition adjustment (R = −0.129 at *p* < 0.01), but positively with these variables for DEs. On the other hand, for DEs, their overall satisfaction with the university was found to be mildly positively correlated with resources and stigma (R = 0.103 at *p* < 0.01), and escape (coping style) (R = 0.079 at *p* < 0.05) (results not shown).

### 3.5. Factors Affecting Student Active Coping and Student Satisfaction with University

Table 9 shows the results of linear regression using the various factors as the independent variables to predict the variance of students’ active coping as the dependent variable. Social connections and coping style (emotional) are significant predictors in both groups. Institutional attributes were significant predictor exclusives to 2yTEs, whereas for DEs, sense of belonging, social adjustment and academic experience and advising were the exclusive significant predictors.

Table 10 shows the results of linear regression using the various factors as the independent variables to predict the variance of students’ satisfaction with the university as the dependent variable. 

## 4. Discussion

This is the first study to investigate DEs and TEs’ experiences of adjusting to university life and factors affecting their active coping and satisfaction with university in an eastern educational context. Our study found that the DEs experienced better adjustment processes and social connections, received more university support, were more likely to use active coping strategies, and felt more satisfied with university than the TEs did. On the flip side, the TEs experienced stigmatization, heavier study loads, less opportunities for overseas exchanges, and received less university resources. Both groups of students considered general support and advising, social adjustment, and institutional attributes to be the factors affecting their overall satisfaction with the university. Sense of belonging was the factor affecting TEs’ overall satisfaction, while academic experience and advising, and active coping were the factors for the DEs. On the other hand, both groups of students considered social connections and emotional coping to be the factors affecting active coping. The factors specifically affecting the TEs’ active coping were institutional attributes, whereas those affecting DEs’ included sense of belonging; social adjustment; overall satisfaction with the university and academic experience; and advising. In the following sections, we will discuss how the findings of this study validated the instrument used, differences between the two groups in terms of their academic and social involvement, the factors affecting overall university satisfaction and active coping for the two groups of students, and then the implications of the study.

### 4.1. Instrument Validation

The results of the study indicate that the HKML-TSQ is applicable to university students (both direct and TEs) in eastern countries, with high Cronbach’s alphas ranging from 0.728 to 0.903 (except for “transition adjustment” with a Cronbach’s alpha of 0.583). For the five constructs used in our study: perceived disparity (transfer vs non-transfer students); adjustment processes; university support; coping style; and social support at the university; the total factor variances ranged from 31.86 to 54.84. Beside CFA, other models such as GFI, CFI, TLI, and RMSEA have been used to test the fit indices, with promising results. The original ML-TSQ was tested on 319 TEs in one university [29]. Perhaps due to the smaller sample size, some factors were not identified in the original study. In this study, most of the items from the original ML-TSQ were retained, but some were modified for the local context. Because of these modifications, it is difficult to make direct comparisons of the constructs. Of the factors identified in the US [29] and in our study, (1) the four-item subscale of active coping was identical; and (2) the subscale of social connections in both contexts consisted of eight items. We also found that the subscales identified between the DEs and TEs were comparable. These similar findings might be due to the fact that the students shared similar cultural backgrounds and received education under the same system and policies. 

### 4.2. Differences of Academic and Social Experience between the Two Groups of Students

Our study findings support that the TEs’ experiences of academic and social integration are generally negative or less desirable than those of the DEs. The finding that the TEs in this study experienced the feeling of stigmatization is consistent with previous studies in the US [40]. Community college students in western countries are demographically more diverse (e.g., a large range of ages) and they have various reasons for enrolling (e.g., financial constraints) [30], whereas community college students in Hong Kong are mainly fresh graduates of senior secondary education with the primary and often the only goal of articulating into university [41]. This finding that the students felt stigmatized for being TEs can be explained contextually by their self-perceptions due to Hong Kong’s education system. A study of Hong Kong community college students’ perceptions of self-worth found that the majority of them considered the education system serves the functions of “differentiation” and “selection” (p. 257) [42] that contrasts with “integration” for academic success in higher education [10]. In a related study, students who were unable to get into university “straight away” after completing secondary education perceived themselves as “losers” (p. 280) [43]. Such a mindset might be rooted in their beliefs and linger on even after they have articulated to university, thus resulting in self-stigmatization that can be mentally unhealthy [44].

On the other hand, TEs bear heavier study loads, most likely due to the long-standing ills of credit transfer [4]. For instance, some credits for subjects studied in their community colleges might not be accepted by their universities, possibly leading not only to heavier course loads but also to delays in graduation [5]. This presents a typical mismatch between the idealistic situation (i.e., all credits successfully transferred) and the reality (e.g., credit loss) that has been shown as a significant predictor of TEs’ transition experiences [2]. According to Tinto’s model of student attrition [21], this obstacle to the TEs’ academic integration can even lead to a higher risk of dropping out [22]. Another finding that TEs perceived themselves as receiving less resources and support than DEs, brings about a three-fold explanation. Without the comprehensive induction or orientation that is sometimes exclusive to DEs [17], TEs are less likely to be aware of university support services such as counselling [12]. At the same time, their lack of awareness of, and thereby access to, these resources and support can also be explained by their hesitation to be proactive in asking for help [15]. This can, in turn, be a consequence of feeling “underprepared” and “unconfident” (p. 4) [45]. Additionally, the perception of university administrators that TEs with prior experience from community colleges can navigate the university environment well [4] further affects the allocation of resources to TEs.

### 4.3. Factors Affecting Students’ Active Coping and Satisfaction with University

We found that academic experience and advising and active coping were the key factors for the DEs but not for the TEs. With the theoretically validated importance of academic integration and involvement in students’ commitment to university studies [12], our study offers an interesting finding that academic experience and advising was not a key factor affecting the TEs’ satisfaction with the university. One of the possible explanations comes from their perceived experience of stigmatization. They might, in advance, have expected less support and thus did not expect much from the university. In addition, their heavy study loads might have overshadowed their expectations to seek advice. Furthermore, as mentioned, the transfer process entails a strong mechanism of screening such that only the best-performing students from community colleges can articulate to universities [4]. This might further lower the likelihood of their requiring academic advising [46].

Rather, sense of belonging was the key factor specifically affecting the TEs’ overall satisfaction. The heavy study load, coupled with the shorter duration of their university courses, might have affected their participation in university activities. Our study results (Table 9) supported that, compared with the DEs, the TEs experienced more difficulties in making friends, participated less in social activities, and had less opportunities for overseas exchange or other types of university support. Without such integration into the academic and social systems of the university, based on our study’s theoretical framework of I-E-O [18], TEs would receive less support for the “environmental variables”. Thus, they would be more likely to have lower levels of commitment to the university [24]. With a poor sense of belonging to the university, as a consequence, TEs have higher dropout rates [7].

On the other hand, both groups of students considered social connections and emotional coping to be the key factors influencing their active coping. Establishing social connections and coping emotionally with a new environment are common adaptive practices for students transitioning into university study [47]. After all, in the context of this study (i.e., Hong Kong), both groups of students shared similar socio-cultural backgrounds and experienced their growth and development under the same education system [48].

The construct of institutional attributes was the only key factor affecting the TEs’ active coping, while sense of belonging, social adjustment, overall satisfaction with the university and academic experience, and advising were factors for the DEs. Before articulating to university, TEs had already spent 2 years in community colleges, half the duration of the 4-year undergraduate study. They might already have become immersed in the culture of the community college and would therefore find it difficult to cope with the new institutional environment (i.e., “campus culture shock”) [15,30]. As critical institutional attributes of universities, large class sizes and impersonal organizational structures might overwhelm TEs [4,40]. Overall, these findings suggest that TEs might feel underprepared and stigmatized, particularly in non-academic aspects, so that they are more likely to adopt active coping to adjust to university study and campus life. In other words, they have to rely on their own planning and efforts (i.e., active coping) to discover their idiosyncratic paths within a limited period of time in university [49].

### 4.4. Implications

The findings of this study have implications for various stakeholders of community colleges and universities, including the management, administrators, academic advisors, student affairs officers, and student bodies. In terms of academic integration, academic advisors, who are often academic staff, should be aware of essential information useful for credit transfer, including TEs’ course loads, the requirements of course selection, and the process of credit transfer. University counsellors should also be notified about the potential of heavy study loads, among other issues (e.g., mental health) encountered by TEs, so that they can be better prepared to assist students in their academic and social adjustment [11]. Excessive study loads also indirectly take away their opportunities to participate in overseas exchanges. While community colleges and universities should continue working hand-in-hand towards improving the system and policies associated with credit transfer [50], the internationalization-at-home (IaH) experience can be introduced to TEs. IaH refers to exposing students to both formal and informal learning experiences via technology-mediated communication [51]. This can serve as an “alternative to student exchange”, with less time [52]. 

On the other hand, TEs’ unique experience of feeling stigmatized about their status is noteworthy. The self-stigmatization might lower their expectations about the amount of campus support and resources they would receive. Nonetheless, from the perspective of service quality assurance [53], the management and administration personnel have the obligation to maintain the equity of access to campus support services for students, regardless of their entry paths. In fact, in order to help TEs towards both academic and social integration, orientation, advising and support services should all be well-provided to welcome and acclimatize them, yet previous studies have criticized current efforts as being inadequate [4]. In addition, student affairs officers can conduct campus visits for incoming TEs before the semester starts, to minimize “unpleasant surprises” (p. 13) [50]. These actions taken by universities could mitigate problems associated with the campus culture shock, which in turn could enhance their sense of belonging and thereby satisfaction with the university [15]. Additionally, to enhance communication and collaboration between students and faculty, representatives of transfer students can be elected to staff-student consultative committees as a formal communication channel between students and the university [54]. Strengthening faculty–student interactions via such channels might also improve students’ sense of belonging [55]. Furthermore, student-run organizations (e.g., student associations or societies) can also play an important role to facilitate the interaction between DEs and TEs and help them adapt to campus life, through different activities such as orientation and campus experience camps [56].

### 4.5. Limitations

After numerous university-wide attempts were made to recruit students using multiple methods and incentives, 12% of all students in the university, including 27% of all TEs, participated in this study. The sample size of more than 840 students in each group, involving all departments, suggests high generalizability of the study’s results to the study university. However, the study’s generalizability to other universities is questionable, given that the university in which this study was conducted contains the largest number of TEs of all the universities in Hong Kong. Future research could involve other universities in Hong Kong to gain a more comprehensive understanding of the two student populations. Besides, the cross-sectional nature of our study creates a limitation that respondents might find it difficult to reflect accurately on their past and current experiences. Thus, longitudinal studies could be adopted so that changes over time could be considered. Furthermore, the construct “transition adjustment” should be interpreted with caution, because of its slightly low internal consistency reliability (Cronbach alpha 0.583). Although the alpha was close to the acceptable level of 0.6–0.7 [57], the inter-relatedness of the items in this construct should be examined further. For instance, the item of “drop in GPAs” might not be applied to DEs who were straight from secondary school, because they did not have GPAs in their secondary schools with which to compare. 

## 5. Conclusions

To the best of our knowledge, this is the first study exploring and comparing the experiences and perceptions of DEs and TEs in their adjustment to the university from both academic and social perspectives. The study found that TEs have relatively less desirable experiences in the adjustment processes than do their direct-entry counterparts. Different stakeholders from community colleges, universities, and student bodies should work collaboratively to improve students’ transitional experiences before, during and after admission to the university.

## Figures and Tables

**Table 1:** Demographics of 2yTEs and DEs.

	2yTEs	DEs	Overall	University-Wide
**Number of students by gender (Percentage to total)**				
Female	541 (64.3%)	659 (67.4%)	1200 (66.0%)	7626 (51.5%)
Male	300 (35.7%)	319 (32.6%)	619 (34.0%)	7173 (48.5%)
Total	841	978	1819	14,799
**Number of students by age**				
Min	20	19	19	17
Max	52	43	52	52
Mean (SD)	22.26 (1.77)	21.09 (1.89)	21.63 (1.92)	21.58 (1.79)

**Table 2:** Factor analysis on perceived disparity.

Perceived Disparity: Transfer vs. Non-Transfer Students (Fixed Factor = 2)		2yTEs	DEs	Overall
	*N*	839	975	1814
	KMO	0.848	0.892	0.877
Resources and sigma	Factor loading	4 items	3 items	4 items
	Variance	36.52	5.70	41.75
	α	0.770	0.830	0.796
Academic study	Factor loading	4 items	5 items	4 items
	Variance	6.07	45.65	5.31
	α	0.695	0.784	0.745
Total Variance		42.59	51.35	47.06

**Table 3:** Factor analysis on adjustment process.

Adjustment Process at University (Fixed Factor = 2)		2yTEs	DEs	Overall
	*N*	840	975	1815
	KMO	0.737	0.766	0.745
Social adjustment	Factor loading	6 items	6 items	6 items
	Variance	22.43	23.44	21.52
	α	0.763	0.747	0.753
Transition adjustment	Factor loading	4 items	4 items	4 items
	Variance	9.43	9.87	10.86
	α	0.513	0.633	0.583
Total Variance		31.86	33.31	32.37

**Table 4:** Factor analysis on university support.

University Support		2yTEs	DEs	Overall
	*N*	411	550	961
	KMO	0.963	0.941	0.948
General support and advising	Factor loading	11 items	14 items	10 items
	Variance	39.11	39.21	39.59
	α	0.890	0.897	0.903
Academic experience and advising	Factor loading	8 items	2 items	6 items
	Variance	4.31	3.26	4.29
	α	0.878	0.853	0.863
Institutional attributes	Factor loading	3 items	6 items	4 items
	Variance	4.07	4.59	3.42
	α	0.685	0.864	0.728
Total Variance		47.49	47.06	47.30

**Table 5:** Factor analysis on coping styles at university.

Coping Styles at University		2yTEs	DEs	Overall
	*N*	841	978	1819
	KMO	0.839	0.840	0.845
Coping style: avoidance	Factor loading	5 items	3 items	3 items
	Variance	25.87	25.36	25.95
	α	0.846	0.875	0.875
Coping style: emotional	Factor loading	/	3 items	3 items
	Variance		3.91	4.15
	α		0.760	0.765
Coping style: active	Factor loading	4 items	6 items	6 items
	Variance	19.54	18.04	18.42
	α	0.820	0.809	0.814
Coping style: active	Factor loading	2 items	/	/
	Variance	4.92		
	α	0.771		
Coping style: escape	Factor loading	4 items	3 items	3 items
	Variance	4.51	6.03	5.12
	α	0.726	0.749	0.741
Total Variance		54.84	53.33	53.63

**Table 6:** Factor analysis on social support at university.

Social Support at University		2yTEs	DEs	Overall
	*N*	840	978	1818
	KMO	0.853	0.871	0.967
Social connections	Factor loading	8 items	8 items	8 items
	Variance	38.43	36.69	38.71
	α	0.728	0.733	0.730
Sense of belonging	Factor loading	2 items	2 items	2 items
	Variance	8.88	10.66	8.45
	α	0.842	0.791	0.817
Total Variance		47.32	47.35	47.16

**Table 7:** Factors and their internal consistencies.

	Cronbach’s Alpha
Perceived disparity: transfer vs non-transfer students	
Resources and stigma (4 items)	0.796
Academic study (4 items)	0.745
Adjustment process at the university	
Social adjustment (6 items)	0.753
Transition adjustment (4 items)	0.583
University support	
General support and advising (10 items)	0.903
Academic experience and advising (6 items)	0.863
Institutional attributes (4 items)	0.728
Coping style at the university	
Coping style: avoidance (3 items)	0.875
Coping style: emotional (3 items)	0.765
Coping style: active (6 items)	0.814
Coping style: escape (3 items)	0.741
Social support at the university	
Social connections (8 items)	0.730
Sense of belonging (2 items)	0.817

**Table 8:** Comparison in experience and perceptions between 2yTEs and DEs.

Factors	Overall	2yTEs	DEs	Sig. *t*-Test
*Perceived disparity: transfer vs non-transfer students ^a^*				
Resources and stigma	2.94 ± 0.72	3.03 ± 0.73	2.87 ± 0.70	**<0.0001**
Academic study	3.17 ± 0.77	3.40 ± 0.77	2.97 ± 0.71	**<0.0001**
*Adjustment process at the university*				
Social adjustment	3.26 ± 0.58	3.23 ± 0.59	3.29 ± 0.56	**0.025**
Transition adjustment	3.07 ± 0.69	3.20 ± 0.67	2.95 ± 0.68	**<0.0001**
*University support*				
General support and advising	2.85 ± 0.58	2.76 ± 0.57	2.92 ± 0.59	**<0.0001**
Academic experience and advising	2.89 ± 0.56	2.85 ± 0.55	2.93 ± 0.56	**0.002**
Institutional attributes	2.90 ± 0.53	2.86 ± 0.54	2.94 ± 0.53	**0.001**
Overall university satisfaction ^b^	2.96 ± 0.63	2.89 ± 0.67	3.01 ± 0.59	**<0.0001**
*Coping style at the university*				
Coping style: avoidance	2.74 ± 0.83	2.73 ± 0.83	2.74 ± 0.83	0.775
Coping style: emotional	3.10 ± 0.72	3.07 ± 0.72	3.12 ± 0.72	0.115
Coping style: active ^c^	3.53 ± 0.55	3.50 ± 0.56	3.55 ± 0.53	**0.042**
Coping style: escape	2.78 ± 0.79	2.78 ± 0.78	2.78 ± 0.80	0.996
*Social support at the university*				
Social connections	3.34 ± 0.54	3.30 ± 0.55	3.37 ± 0.53	**0.008**
Sense of belonging	3.31 ± 0.81	3.28 ± 0.85	3.34 ± 0.77	0.083

^a^: The higher the score, the more uneven between direct and 2yCCT for perceived disparity: transfer vs non-transfer students; ^b^: Single item and the dependent variable of the study; ^c^: dependent variable of the study. Data in bold: significant results.

**Table 9:** Results of Linear Regression for predicting students’ active coping.

	2yTEs	DEs	Overall
F(df)	F(3, 755) = 93.874	F(6, 905) = 65.813	F(5, 1665) = 131.138
R^2^	0.272	0.304	0.283
Sig.	*p* < 0.001	*p* < 0.001	*p* < 0.001
Significant Predictors	*Social connections* (β = 0.366, *p* < 0.001) *Coping style: emotional* (β = 0.204, *p* < 0.001) *Institutional attributes* (β = 0.147, *p* < 0.001)	*Social connections* (β = 0.257, *p* < 0.001) *Coping style: emotional* (β = 0.141, *p* < 0.001) *Sense of belonging* (β = 0.131, *p* < 0.001) *Social adjustment* (β = 0.101, *p* = 0.003) *Overall satisfaction with the university* (β = 0.095, *p* = 0.005) *Academic experience and advising* (β = 0.081, *p* = 0.019)	*Social connections* (β = 0.314, *p* < 0.001) *Coping style: emotional* (β = 0.150, *p* < 0.001) *Institutional attributes* (β = 0.115, *p* < 0.001) *Sense of belonging* (β = 0.112, *p* < 0.001) *Overall satisfaction with the university* (β = 0.079, *p* = 0.002)

**Table 10:** Results of Linear Regression for predicting student overall satisfaction with university.

	2yTEs	DEs	Overall
F(df)	F(4, 752) = 151.017	F(5, 906) = 121.429	F(7, 1662) = 176.970
R^2^	0.445	0.401	0.427
Sig.	*p* < 0.001	*p* < 0.001	*p* < 0.001
Significant Predictors	*Institutional attributes* (β = 0.329, *p* < 0.001) *General support and advising* (β = 0.275, *p* < 0.001) *Sense of belonging* (β = 0.129, *p* < 0.001) *Social adjustment* (β = 0.103, *p* = 0.002)	*Institutional attributes* (β = 0.237, *p* < 0.001) *General support and advising* (β = 0.187, *p* < 0.001) *Academic experience and advising* (β = 0.178, *p* < 0.001) *Social adjustment* (β = 0.136, *p* < 0.001) *Coping style: active* (β = 0.098, *p* = 0.001)	*Institutional attributes* (β = 0.278, *p* < 0.001) *General support and advising* (β = 0.216, *p* < 0.001) *Social adjustment* (β = 0.125, *p* < 0.001) *Academic experience and advising* (β = 0.110, *p* < 0.001) *Sense of belonging* (β = 0.075, *p* = 0.001) *Academic study* (β = −0.049, *p* = 0.009) *Coping style: active* (β = 0.048, *p* = 0.020)
